# Supplementary material for: NEURO-COVAX: An Italian Population-Based Study of Neurological Complications after COVID-19 Vaccinations
Source: Vaccines (Basel). 2023 Oct 21;11(10):1621. doi: 10.3390/vaccines11101621 (PMC10610846; doi:10.3390/vaccines11101621)
Supplement: Supplementary file 1 [file vaccines-11-01621-s001.zip › Salsone M et al., Supplematary NEURO-COVAX QUESTIONNAIRE.pdf]

I.R.C.C.S. Ospedale San Raffaele
Gruppo San Donato

This study was approved by the National Ethical Committee Spallanzani, National Institute for Infectious Diseases Lazzaro Spallanzani, Rome, Italy under the project ID Number 362

NEURO-COVAX QUESTIONNARIE

FIRST DOSE-Acute

SECTION I- VACCINATION INFORMATION
Vaccine center:
Date of administration:
Vaccine:

SECTION II-PERSONAL DATA
Surname and name:
Date of birth:
Telephone number/e-mail address and residence:

SECTION III: NEUROLOGICAL COMPLICATION LIST
Did you experience neurological symptoms after receiving the vaccine?

☐ None
☐ If yes, which of the following symptoms (indicate one or more symptoms)
- ☐ Vertigo
- ☐ Headache
- ☐ Muscle pain
- ☐ Muscle spasms
- ☐ Paresthesias (tingling and sensitivity changes)
- ☐ Tremor
- ☐ Double vision
- ☐ Tinnitus (ringing in the ears)
- ☐ Dysphonia (voice changes)
- ☐ Excessive daytime sleepiness
- ☐ Other

SECTION IV: NEUROLOGICAL COMPLICATION CHARACTERIZZATION (SYMPTOM BOX)
Indicate for each of the symptoms in the relevant section, the appearance and duration.

**Vertigo**
When did they appear?<br>☐ in the first 15 minutes ☐ after 15 minutes
How long did they last?<br>☐ 5-10 minutes ☐ more than 10 minutes

**Headache**

When did they appear?
☐ in the first 15 minutes ☐ after 15 minutes

How long did they last?
☐ 5-10 minutes ☐ more than 10 minutes

Muscle Pain

When did they appear?
☐ in the first 15 minutes ☐ after 15 minutes

How long did they last?
☐ 5-10 minutes ☐ more than 10 minutes

Muscle Spasm

When did they appear?
☐ in the first 15 minutes ☐ after 15 minutes

How long did they last?
☐ 5-10 minutes ☐ more than 10 minutes

Paresthesies (tingling and sensitivity changes)

When did they appear?
☐ in the first 15 minutes ☐ after 15 minutes

How long did they last?
☐ 5-10 minutes ☐ more than 10 minutes

Tremor

When did they appear?
☐ in the first 15 minutes ☐ after 15 minutes

How long did they last?
☐ 5-10 minutes ☐ more than 10 minutes

Diplopia

When did they appear?
☐ in the first 15 minutes ☐ after 15 minutes

How long did they last?
☐ 5-10 minutes ☐ more than 10 minutes

Tinnitus (ringing in the ears)

When did they appear?
☐ in the first 15 minutes ☐ after 15 minutes

How long did they last?<br>☐ 5-10 minutes ☐ more than 10 minutes

**Dysphonia (alteration of the voice)**
When did they appear?<br>☐ in the first 15 minutes ☐ after 15 minutes
How long did they last?<br>☐ 5-10 minutes ☐ more than 10 minutes

**Excessive daytime sleepiness**
When did they appear?<br>☐ in the first 15 minutes ☐ after 15 minutes
How long did they last?<br>☐ 5-10 minutes ☐ more than 10 minutes

**Other**
When did they appear?<br>☐ in the first 15 minutes ☐ after 15 minutes
How long did they last?<br>☐ 5-10 minutes ☐ more than 10 minutes

Date ____

Signature ____

NEURO-COVAX QUESTIONNARIE

FIRST DOSE-Subacute

SECTION I- VACCINATION INFORMATION

Vaccine center:
Date of administration:
Vaccine:

SECTION II-PERSONAL DATA

Surname and name:
Date of birth:
Telephone number/e-mail address and residence:

SECTION III: NEUROLOGICAL COMPLICATION LIST

Did you experience neurological symptoms after receiving the vaccine?

☐ None
☐ If yes, which of the following symptoms (indicate one or more symptoms)
- ☐ Vertigo
- ☐ Headache
- ☐ Muscle pain
- ☐ Muscle spasms
- ☐ Paresthesias (tingling and sensitivity changes)
- ☐ Tremor
- ☐ Double vision
- ☐ Tinnitus (ringing in the ears)
- ☐ Dysphonia (voice changes)
- ☐ Excessive daytime sleepiness
- ☐ Insomnia
- ☐ Cognitive Fog
- ☐ Smell alterations
- ☐ Taste alterations
- ☐ Other

SECTION IV: NEUROLOGICAL COMPLICATION CHARACTERIZZATION (SYMPTOM BOX)

Indicate for each of the symptoms in the relevant section, the appearance and duration.

**Vertigo**
When did they appear?<br>☐ in the first hours ☐ in the first 3 days ☐ from the 4th to the 7th day ☐ from the 8th to the 14th day
How long did they last?<br>☐ less than a day ☐ up to a week ☐ over a week

**Headache**
When did they appear?<br>☐ in the first hours ☐ in the first 3 days ☐ from the 4th to the 7th day ☐ from the 8th to the 14th day

How long did they last?
☐ less than a day ☐ up to a week ☐ over a week

Muscle Pain

When did they appear?
☐ in the first hours ☐ in the first 3 days ☐ from the 4th to the 7th day ☐ from the 8th to the 14th day

How long did they last?
☐ less than a day ☐ up to a week ☐ over a week

Muscle Spasm

When did they appear?
☐ in the first hours ☐ in the first 3 days ☐ from the 4th to the 7th day ☐ from the 8th to the 14th day

How long did they last?
☐ less than a day ☐ up to a week ☐ over a week

Paresthesies (tingling and sensitivity changes)

When did they appear?
☐ in the first hours ☐ in the first 3 days ☐ from the 4th to the 7th day ☐ from the 8th to the 14th day

How long did they last?
☐ less than a day ☐ up to a week ☐ over a week

Tremor

When did they appear?
☐ in the first hours ☐ in the first 3 days ☐ from the 4th to the 7th day ☐ from the 8th to the 14th day

How long did they last?
☐ less than a day ☐ up to a week ☐ over a week

Diplopia

When did they appear?
☐ in the first hours ☐ in the first 3 days ☐ from the 4th to the 7th day ☐ from the 8th to the 14th day

How long did they last?
☐ less than a day ☐ up to a week ☐ over a week

Tinnitus (ringing in the ears)

How long did they last?
☐ less than a day ☐ up to a week ☐ over a week

How long did they last?
☐ less than a day ☐ up to a week ☐ over a week

Dysphonia (alteration of the voice)

When did they appear?
☐ in the first hours ☐ in the first 3 days ☐ from the 4th to the 7th day ☐ from the 8th to the 14th day

How long did they last?
☐ less than a day ☐ up to a week ☐ over a week

Excessive daytime sleepiness

When did they appear?
☐ in the first hours ☐ in the first 3 days ☐ from the 4th to the 7th day ☐ from the 8th to the 14th day

How long did they last?
☐ less than a day ☐ up to a week ☐ over a week

Insomnia

When did they appear?
☐ in the first hours ☐ in the first 3 days ☐ from the 4th to the 7th day ☐ from the 8th to the 14th day

How long did they last?
☐ less than a day ☐ up to a week ☐ over a week

Cognitive Fog

When did they appear?
☐ in the first hours ☐ in the first 3 days ☐ from the 4th to the 7th day ☐ from the 8th to the 14th day

How long did they last?
☐ less than a day ☐ up to a week ☐ over a week

**Smell Alterations**
When did they appear?<br>☐ in the first hours ☐ in the first 3 days ☐ from the 4th to the 7th day ☐ from the 8th to the 14th day
How long did they last?<br>☐ less than a day ☐ up to a week ☐ over a week

**Taste Alterations**
When did they appear?<br>☐ in the first hours ☐ in the first 3 days ☐ from the 4th to the 7th day ☐ from the 8th to the 14th day
How long did they last?<br>☐ less than a day ☐ up to a week ☐ over a week

**Other**
When did they appear?<br>☐ in the first hours ☐ in the first 3 days ☐ from the 4th to the 7th day ☐ from the 8th to the 14th day
How long did they last?<br>☐ less than a day ☐ up to a week ☐ over a week

Date ____

Signature ____

NEURO-COVAX QUESTIONNARIE
SECOND DOSE-Acute

SECTION I- VACCINATION INFORMATION
Vaccine center:
Date of administration:
Vaccine:

SECTION II-PERSONAL DATA
Surname and name:
Date of birth:
Telephone number/e-mail address and residence:

SECTION III: NEUROLOGICAL COMPLICATION LIST
Did you experience neurological symptoms after receiving the vaccine?
☐ None
☐ If yes, which of the following symptoms (indicate one or more symptoms)
- ☐ Vertigo
- ☐ Headache
- ☐ Muscle pain
- ☐ Muscle spasms
- ☐ Paresthesias (tingling and sensitivity changes)
- ☐ Tremor
- ☐ Double vision
- ☐ Tinnitus (ringing in the ears)
- ☐ Dysphonia (voice changes)
- ☐ Excessive daytime sleepiness
- ☐ Other

SECTION IV: NEUROLOGICAL COMPLICATION CHARACTERIZZATION (SYMPTOM BOX)
Indicate for each of the symptoms in the relevant section, the appearance and duration.

**Vertigo**
When did they appear?<br>☐ in the first 15 minutes ☐ after 15 minutes
How long did they last?<br>☐ 5-10 minutes ☐ more than 10 minutes

**Headache**
When did they appear?<br>☐ in the first 15 minutes ☐ after 15 minutes
How long did they last?

☐ 5-10 minutes ☐ more than 10 minutes

Muscle Pain

When did they appear?
☐ in the first 15 minutes ☐ after 15 minutes

How long did they last?
☐ 5-10 minutes ☐ more than 10 minutes

Muscle Spasm

When did they appear?
☐ in the first 15 minutes ☐ after 15 minutes

How long did they last?
☐ 5-10 minutes ☐ more than 10 minutes

Paresthesies (tingling and sensitivity changes)

When did they appear?
☐ in the first 15 minutes ☐ after 15 minutes

How long did they last?
☐ 5-10 minutes ☐ more than 10 minutes

Tremor

When did they appear?
☐ in the first 15 minutes ☐ after 15 minutes

How long did they last?
☐ 5-10 minutes ☐ more than 10 minutes

Diplopia

When did they appear?
☐ in the first 15 minutes ☐ after 15 minutes

How long did they last?
☐ 5-10 minutes ☐ more than 10 minutes

Tinnitus (ringing in the ears)

When did they appear?
☐ in the first 15 minutes ☐ after 15 minutes

How long did they last?
☐ 5-10 minutes ☐ more than 10 minutes

Dysphonia (alteration of the voice)

When did they appear?<br>☐ in the first 15 minutes ☐ after 15 minutes
How long did they last?<br>☐ 5-10 minutes ☐ more than 10 minutes

**Excessive daytime sleepiness**
When did they appear?<br>☐ in the first 15 minutes ☐ after 15 minutes
How long did they last?<br>☐ 5-10 minutes ☐ more than 10 minutes

**Other**
When did they appear?<br>☐ in the first 15 minutes ☐ after 15 minutes
How long did they last?<br>☐ 5-10 minutes ☐ more than 10 minutes

Date ____

Signature ____

NEURO-COVAX QUESTIONNARIE
SECOND DOSE-Subacute

SECTION I- VACCINATION INFORMATION
Vaccine center:
Date of administration:
Vaccine:

SECTION II-PERSONAL DATA
Surname and name:
Date of birth:
Telephone number/e-mail address and residence:

SECTION III: NEUROLOGICAL COMPLICATION LIST
Did you experience neurological symptoms after receiving the vaccine?
☐ None
☐ If yes, which of the following symptoms (indicate one or more symptoms)
- ☐ Vertigo
- ☐ Headache
- ☐ Muscle pain
- ☐ Muscle spasms
- ☐ Paresthesias (tingling and sensitivity changes)
- ☐ Tremor
- ☐ Double vision
- ☐ Tinnitus (ringing in the ears)
- ☐ Dysphonia (voice changes)
- ☐ Excessive daytime sleepiness
- ☐ Insomnia
- ☐ Cognitive Fog
- ☐ Smell alterations
- ☐ Taste alterations
- ☐ Other

SECTION IV: NEUROLOGICAL COMPLICATION CHARACTERIZZATION (SYMPTOM BOX)
Indicate for each of the symptoms in the relevant section, the appearance and duration.

**Vertigo**
When did they appear?<br>☐ in the first hours ☐ in the first 3 days ☐ from the 4th to the 7th day ☐ from the 8th to the 14th day
How long did they last?<br>☐ less than a day ☐ up to a week ☐ over a week

**Headache**

When did they appear?
☐ in the first hours ☐ in the first 3 days ☐ from the 4th to the 7th day ☐ from the 8th to the 14th day

How long did they last?
☐ less than a day ☐ up to a week ☐ over a week

Muscle Pain

When did they appear?
☐ in the first hours ☐ in the first 3 days ☐ from the 4th to the 7th day ☐ from the 8th to the 14th day

How long did they last?
☐ less than a day ☐ up to a week ☐ over a week

Muscle Spasm

When did they appear?
☐ in the first hours ☐ in the first 3 days ☐ from the 4th to the 7th day ☐ from the 8th to the 14th day

How long did they last?
☐ less than a day ☐ up to a week ☐ over a week

Paresthesies (tingling and sensitivity changes)

When did they appear?
☐ in the first hours ☐ in the first 3 days ☐ from the 4th to the 7th day ☐ from the 8th to the 14th day

How long did they last?
☐ less than a day ☐ up to a week ☐ over a week

Tremor

When did they appear?
☐ in the first hours ☐ in the first 3 days ☐ from the 4th to the 7th day ☐ from the 8th to the 14th day

How long did they last?
☐ less than a day ☐ up to a week ☐ over a week

Diplopia

When did they appear?<br>☐ in the first hours ☐ in the first 3 days ☐ from the 4th to the 7th day ☐ from the 8th to the 14th day
How long did they last?<br>☐ less than a day ☐ up to a week ☐ over a week

**Tinnitus (ringing in the ears)**
How long did they last?<br>☐ less than a day ☐ up to a week ☐ over a week
How long did they last?<br>☐ less than a day ☐ up to a week ☐ over a week

**Dysphonia (alteration of the voice)**
When did they appear?<br>☐ in the first hours ☐ in the first 3 days ☐ from the 4th to the 7th day ☐ from the 8th to the 14th day
How long did they last?<br>☐ less than a day ☐ up to a week ☐ over a week

**Excessive daytime sleepiness**
When did they appear?<br>☐ in the first hours ☐ in the first 3 days ☐ from the 4th to the 7th day ☐ from the 8th to the 14th day
How long did they last?<br>☐ less than a day ☐ up to a week ☐ over a week

**Insomnia**
When did they appear?<br>☐ in the first hours ☐ in the first 3 days ☐ from the 4th to the 7th day ☐ from the 8th to the 14th day
How long did they last?<br>☐ less than a day ☐ up to a week ☐ over a week

**Cognitive Fog**
When did they appear?

☐ in the first hours ☐ in the first 3 days ☐ from the 4th to the 7th day ☐ from the 8th to the 14th day

How long did they last?
☐ less than a day ☐ up to a week ☐ over a week

Smell Alterations

When did they appear?
☐ in the first hours ☐ in the first 3 days ☐ from the 4th to the 7th day ☐ from the 8th to the 14th day

How long did they last?
☐ less than a day ☐ up to a week ☐ over a week

Taste Alterations

When did they appear?
☐ in the first hours ☐ in the first 3 days ☐ from the 4th to the 7th day ☐ from the 8th to the 14th day

How long did they last?
☐ less than a day ☐ up to a week ☐ over a week

Other

When did they appear?
☐ in the first hours ☐ in the first 3 days ☐ from the 4th to the 7th day ☐ from the 8th to the 14th day

How long did they last?
☐ less than a day ☐ up to a week ☐ over a week

Date ____

Signature ____
