# Supplementary material for: NEURO-COVAX: An Italian Population-Based Study of Neurological Complications after COVID-19 Vaccinations
Source: Vaccines (Basel). 2023 Oct 21;11(10):1621. doi: 10.3390/vaccines11101621 (PMC10610846; doi:10.3390/vaccines11101621)
Supplement: Supplementary file 1 [file vaccines-11-01621-s001.zip › Supplementary Table S1.pdf]

TABLE S1. Defining onset and duration of neurological complications in the symptomatic group vaccinated in the massive Hub Novegro (Lombardy) between 7 July 2021 and 16 July 2021, distinguished according to the dose

NEUROLOGICAL COMPLICATION	*I DOSE(n)*				*II DOSE(n)*			
	BNT162b2	mRNA-1273	ChAdOx1nCov-19	*Total*	BNT162b2	mRNA-1273	ChAdOx1nCov-19	*Total*
**HEADACHE**								
**Onset**								
**Acute**								
-within 15 minutes	141	21	20	182	136	30	9	175
-after 15 minutes	1039	161	144	**1344**	81	20	8	109
**Subacute**								
- first hours	720	111	104	935	99	24	13	136
- first 3 days	827	173	109	1109	177	36	11	**224**
- from the 4th to the 7th day	55	5	5	65	7	1	3	11
- from the 8th to the 14th day	34	5	3	42	7	-	3	10
**Duration**								
-<10 minutes	171	25	11	207	73	11	2	86
->10 minutes, less than a day	1032	160	157	1349	147	39	15	201
- a day	1105	189	149	**1443**	187	35	21	**243**
-up to a week	460	98	61	619	93	25	8	126
-more than a week	80	8	24	112	10	1	1	2

NEUROLOGICAL COMPLICATION	*I DOSE (n)*				*II DOSE(n)*			
	BNT162b2	mRNA-1273	ChAdOx1nCov19	*Total*	BNT162b2	mRNA-1273	ChAdOx1nCov-19	*Total*
**EXCESSIVE DAYTIME SLEEPINESS**								
**Onset**								
**Acute**								
-within 15 minutes	47	4	5	56	20	4	2	26
-after 15 minutes	745	129	73	**947**	39	6	4	49
**Subacute**								
- first hours	542	86	58	686	67	14	6	87
- first 3 days	687	119	58	864	129	22	7	**158**
- from the 4th to the 7th day	44	6	1	51	6	-	-	6
- from the 8th to the 14th day	29	2	5	36	4	-	5	9
**Duration**								
-<10 minutes	67	4	7	78	6	1	-	7
->10 minutes, less than a day	499	94	44	637	53	9	6	68
- a day	702	108	68	878	90	18	6	114
-up to a week	770	130	73	**973**	98	15	4	**117**
-more than a week	110	11	11	132	18	3	3	24

NEUROLOGICAL COMPLICATION	*I DOSE(n)*				*II DOSE(n)*			
	BNT162b2	mRNA-1273	ChAdOx1nCov-19	*Total*	BNT162b2	mRNA-1273	ChAdOx1nCov-19	*Total*
**VERTIGO**								
**Onset**								
**Acute**								
-within 15 minutes	158	25	9	192	89	23	10	**122**
-after 15 minutes	236	45	30	**311**	36	2	5	43
**Subacute**								
- first hours	142	33	16	191	12	3	1	16
- first 3 days	133	22	16	171	13	2	2	17
- from the 4th to the 7th day	22	1	1	24	1	-	1	2
- from the 8th to the 14th day	15	6	1	22	2	-	-	2
**Duration**								
-<10 minutes	199	32	9	240	69	12	8	**89**
->10 minutes, less than a day	195	36	30	261	56	13	7	76
- a day	199	42	21	**262**	14	2	3	19
-up to a week	84	19	16	109	10	3	1	14
-more than a week	31	2	7	40	5	-	-	5

NEUROLOGICAL COMPLICATION	*I DOSE(n)*				*II DOSE(n)*			
	BNT162b2	mRNA-1273	ChAdOx1nCov-19	*Total*	BNT162b2	mRNA-1273	ChAdOx1nCov-19	*Total*
**PARESTHESIAS**								
**Onset**								
**Acute**								
-within 15 minutes	133	25	7	165	113	18	6	**137**
-after 15 minutes	124	34	11	**169**	27	9	-	36
**Subacute**								
- first hours	134	25	3	162	12	2	1	15
- first 3 days	80	3	5	88	17	7	1	25
- from the 4th to the 7th day	13	3	1	17	2	-	-	2
- from the 8th to the 14th day	8	2	3	13	1	-	-	1
**Duration**								
-<10 minutes	82	13	3	98	63	5	70	138
->10 minutes, less than a day	175	46	15	**236**	77	22	103	**202**
- a day	136	27	5	168	16	5	22	43
-up to a week	67	24	4	95	13	4	18	35
-more than a week	43	4	3	50	4	-	4	8

NEUROLOGICAL COMPLICATION	*I DOSE(n)*				*II DOSE(n)*			
	BNT162b2	mRNA-1273	ChAdOx1nCov-19	*Total*	BNT162b2	mRNA-1273	ChAdOx1nCov-19	*Total*
**COGNITIVE FOG**								
**Onset**								
**Acute**								
-within 15 minutes	-	-	-	-	-	-	-	-
-after 15 minutes	-	-	-	-	-	-	-	-
**Subacute**								
- first hours	87	14	7	108	15	3	-	18
- first 3 days	136	21	5	**162**	29	3	-	**32**
- from the 4th to the 7th day	20	2	2	24	2	-	1	3
- from the 8th to the 14th day	14	-	3	17	5	-	-	5
**Duration**								
-<10 minutes	-	-	-	-	-	1	-	1
->10 minutes, less than a day	-	-	-	-	1	-	-	1
- a day	99	15	12	126	23	2	-	25
-up to a week	120	17	3	**140**	27	3	1	**31**
-more than a week	41	6	3	50	8	1	-	9

NEUROLOGICAL COMPLICATION	*I DOSE(n)*				*II DOSE(n)*			
	BNT162b2	mRNA-1273	ChAdOx1nCov-19	*Total*	BNT162b2	mRNA-1273	ChAdOx1nCov-19	*Total*
**INSOMNIA**								
**Onset**								
**Acute**								
-within 15 minutes	-	-	.	-	-	-	-	-
-after 15 minutes	-	-	-	-	-	-	-	-
**Subacute**								
- first hours	63	6	9	78	17	2	2	21
- first 3 days	114	11	15	**140**	23	3	5	**31**
- from the 4th to the 7th day	16	2	1	19	1	-	-	1
- from the 8th to the 14th day	10	-	1	11	1	-	-	1
**Duration**								
-<10 minutes	-	-	-	-	-	-	-	-
->10 minutes, less than a day	3	-	2	5	-	-	1	1
- a day	84	10	7	101	10	2	4	16
-up to a week	93	8	13	**114**	26	3	2	**31**
-more than a week	29	1	6	36	6	-	1	7

NEUROLOGICAL COMPLICATION	*I DOSE(n)*				*II DOSE(n)*			
	BNT162b2	mRNA-1273	ChAdOx1nCov-19	*Total*	BNT162b2	mRNA-1273	ChAdOx1nCov-19	*Total*
**TREMOR**								
**Onset**								
**Acute**								
-within 15 minutes	19	3	3	25	2	-	1	3
-after 15 minutes	50	18	40	**108**	1	-	2	3
**Subacute**								
- first hours	42	14	48	104	10	4	1	15
- first 3 days	36	17	21	74	23	9	-	**32**
- from the 4th to the 7th day	-	1	-	1	1	-	-	1
- from the 8th to the 14th day	2	1	1	4	-	-	-	-
**Duration**								
-<10 minutes	22	8	4	34	1	-	1	2
->10 minutes, less than a day	48	13	39	100	2	-	2	4
- a day	60	27	64	**151**	28	13	1	**42**
-up to a week	16	5	7	28	4	1	-	5
-more than a week	5	2	1	8	2	-	-	2

NEUROLOGICAL COMPLICATION	*I DOSE(n)*				*II DOSE(n)*			
	BNT162b2	mRNA-1273	ChAdOx1nCov-19	*Total*	BNT162b2	mRNA-1273	ChAdOx1nCov-19	*Total*
**MUSCLE SPASMS**								
**Onset**								
**Acute**								
-within 15 minutes	23	3	3	29	3	1	-	4
-after 15 minutes	82	18	23	**123**	3	1	1	5
**Subacute**								
- first hours	43	7	12	62	13	3	3	19
- first 3 days	52	12	11	75	12	5	3	**20**
- from the 4th to the 7th day	7	-	1	8	2	-	-	2
- from the 8th to the 14th day	11	3	1	15	2	-	-	2
**Duration**								
-<10 minutes	31	4	3	38	3	-	-	3
->10 minutes, less than a day	81	17	23	**121**	2	2	1	5
- a day	45	9	12	66	16	4	1	**21**
-up to a week	48	8	11	67	10	3	-	13
-more than a week	21	5	3	29	3	1	1	5

NEUROLOGICAL COMPLICATION	*I DOSE(n)* BNT162b2	mRNA-1273	ChAdOx1nCov-19	*Total*	*II DOSE(n)* BNT162b2	mRNA-1273	ChAdOx1nCov-19	*Total*
**DIPLOPIA**								
**Onset**								
**Acute**								
-within 15 minutes	24	7	3	34	16	15	-	**31**
-after 15 minutes	27	5	3	**35**	8	2	-	10
**Subacute**								
- first hours	27	4	-	31	2	1	1	4
- first 3 days	27	3	2	32	9	1	-	10
- from the 4th to the 7th day	3	-	2	5	1	-	-	1
- from the 8th to the 14th day	3	-	1	4	-	-	-	-
**Duration**								
-<10 minutes	26	3	8	37	4	4	1	9
->10 minutes, less than a day	25	3	10	38	2	1	2	5
- a day	33	5	5	**38**	6	2	3	**11**
-up to a week	19	2	4	23	5	-	-	5
-more than a week	9	-	2	11	1	-	-	1

NEUROLOGICAL COMPLICATION	*I DOSE(n)*				*II DOSE(n)*			
	BNT162b2	mRNA-1273	ChAdOx1nCov-19	*Total*	BNT162b2	mRNA-1273	ChAdOx1nCov-19	*Total*
**TINNITUS**								
**Onset**								
**Acute**								
-within 15 minutes	11	3	1	15	5	-	-	5
-after 15 minutes	25	2	4	**31**	4	3	-	**7**
**Subacute**								
- first hours	17	-	2	19	3	2	1	6
- first 3 days	17	2	7	26	2	1	2	5
- from the 4th to the 7th day	11	1	1	13	1	-	1	2
- from the 8th to the 14th day	10	2	1	13	-	-	-	1
**Duration**								
-<10 minutes	18	1	1	20	5	1	-	6
->10 minutes, less than a day	19	4	4	27	4	2	-	6
- a day	25	1	2	**28**	5	3	2	**10**
-up to a week	18	2	7	27	1	-	1	2
-more than a week	11	7	3	21	4	-	-	4

NEUROLOGICAL COMPLICATION	*I DOSE(n)*				*II DOSE(n)*			
	BNT162b2	mRNA-1273	ChAdOx1nCov-19	*Total*	BNT162b2	mRNA-1273	ChAdOx1nCov-19	*Total*
**TASTE ALTERATION**								
**Onset**								
**Acute**								
-within 15 minutes	-	-	-		-	-	-	
-after 15 minutes	-	-	-		-	-	-	
**Subacute**								
- first hours	8	2	-	10	2	-	-	2
- first 3 days	14	7	2	**23**	3	2	-	**5**
- from the 4th to the 7th day	5	1	1	7	-	-	-	-
- from the 8th to the 14th day	14	0	-	4	-	-	-	-
**Duration**								
-<10 minutes	-							
->10 minutes, less than a day	-							
- a day	9	2	-	11	1	-	-	1
-up to a week	13	4	3	**20**	3	2	-	**5**
-more than a week	9	1	1	11	2	-	-	2

NEUROLOGICAL COMPLICATION	*I DOSE(n)*				*II DOSE(n)*			
	BNT162b2	mRNA-1273	ChAdOx1nCov-19	*Total*	BNT162b2	mRNA-1273	ChAdOx1nCov-19	*Total*
**DISPHONIA**								
**Onset**								
**Acute**								
-within 15 minutes	3	-	-	3	2	-	-	2
-after 15 minutes	11	2	1	14	2	1	-	3
**Subacute**								
- first hours	6	1	-	7	3	-	-	3
- first 3 days	11	4	-	**15**	4	-	-	**4**
- from the 4th to the 7th day	3	-	-	3	3	-	-	3
- from the 8th to the 14th day	3	-	-	3	-	-	-	-
**Duration**								
-<10 minutes	5	-	-	5	2	-	-	2
->10 minutes, less than a day	10	2	1	**13**	2	-	-	2
- a day	11	1	-	12	7	1	-	**8**
-up to a week	7	4	1	12	1	-	-	1
-more than a week	5	-	-	5	2	-	-	2

NEUROLOGICAL COMPLICATION	*I DOSE(n)*				*II DOSE(n)*			
	BNT162b2	mRNA-1273	ChAdOx1nCov-19	*Total*	BNT162b2	mRNA-1273	ChAdOx1nCov-19	*Total*
**SMELL ALTERATION**								
**Onset**								
**Acute**								
-within 15 minutes	-	-	-	-	-	-	-	-
-after 15 minutes	-	-	-	-	-	-	-	**-**
**Subacute**								
- first hours	4	2	-	5	2	-	2	4
- first 3 days	11	3	2	**16**	2	-	2	**4**
- from the 4th to the 7th day	-	1	1	2	-	-	-	-
- from the 8th to the 14th day	2	-	-	2	-	-	-	-
**Duration**								
-<10 minutes								
->10 minutes, less than a day	-	-	-	**-**	-	-	-	
- a day	5	1	-	6	-	-	-	
-up to a week	5	2	-	7	3	3	-	**6**
-more than a week	7	3	-	**10**	2	1	-	3
