# Supplementary figures and images for: NEURO-COVAX: An Italian Population-Based Study of Neurological Complications after COVID-19 Vaccinations
Source: Vaccines (Basel). 2023 Oct 21;11(10):1621. doi: 10.3390/vaccines11101621 (PMC10610846; doi:10.3390/vaccines11101621)

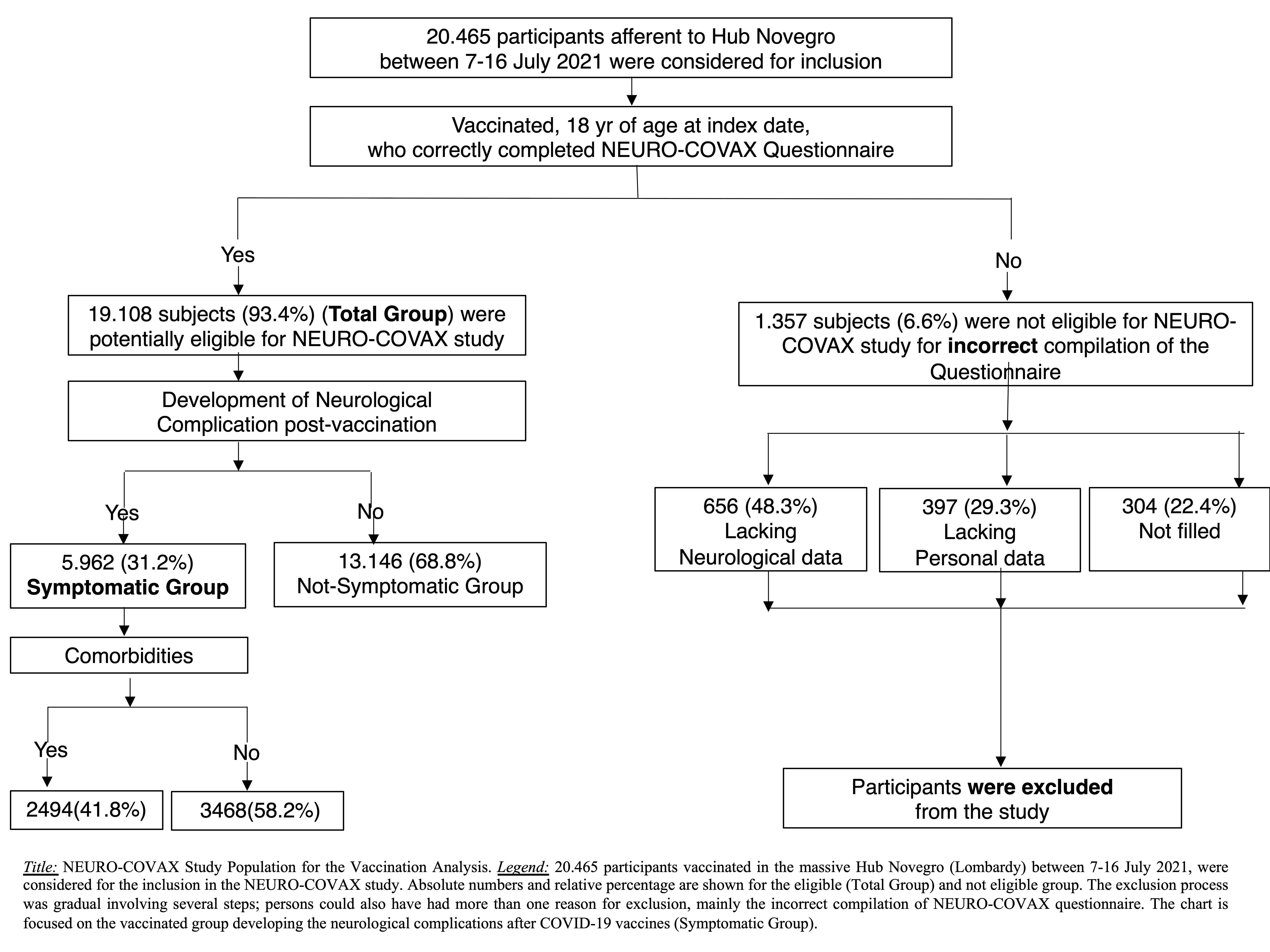

Supplement: Supplementary file 1 [file vaccines-11-01621-s001.zip › New Supplementary Figure 1.jpg]

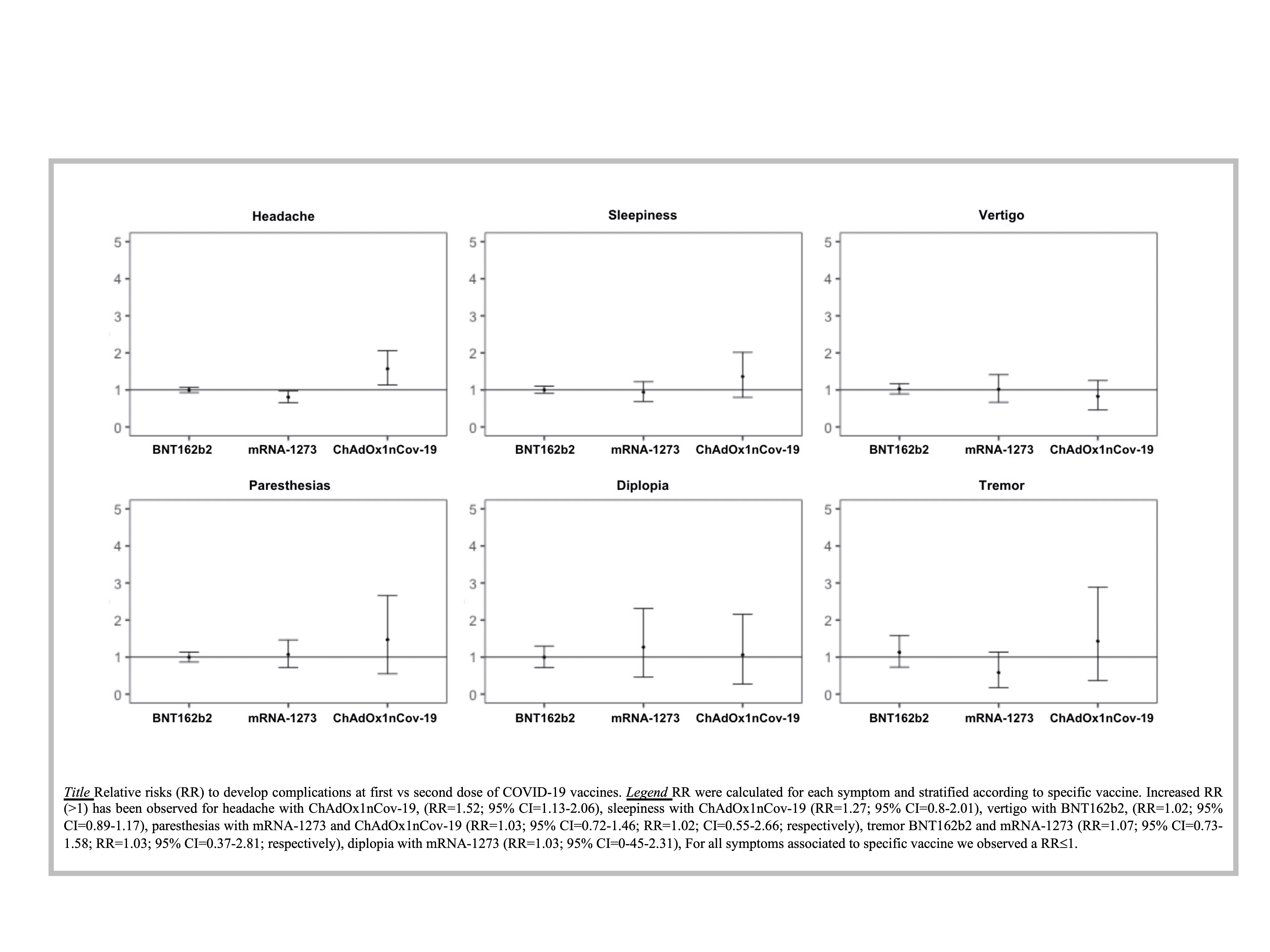

Supplement: Supplementary file 1 [file vaccines-11-01621-s001.zip › Supplemetary Figure 2.jpg]
